# Clinical and epidemiological profile of patients with hereditary angioedema treated in a referral outpatient clinic in Vitória, Espírito Santo - Brazil

**DOI:** 10.1186/1939-4551-8-S1-A230

**Published:** 2015-04-08

**Authors:** Rafael Cicconi Arantes, Débora Martins Ferreira, Murilo Andrade Santana, Faradiba Serpa, Marina Moura Lopes Pereira, Therezinha Ribeiro Moyses, Fernanda Lugão Campinhos

**Affiliations:** 1 Escola Superior De Ciências Da Santa Casa De Misericórdia De Vitória, Brazil

## Background

To assess epidemiological, social and clinical features of patients treated for hereditary angioedema in a referral outpatient clinic at Hospital Santa Casa de Misericordia de Vitoria, ES.

## Methods

An observational, descriptive, cross-sectional study, based on a clinical-epidemiological survey of 51 patients with confirmed diagnosis of hereditary angioedema (HAE) from April 2011 to June 2014. Diagnostic confirmation was through the measurement of C4 and C1 inhibitor (C1-INH) quantitative and functional.

## Results

Data from 51 patients, 29 (57%) females and 22 (43%) males, from 5 to 88 years old (mean: 32 years) was evaluated. Patients belonged to 7 families, 20 of them from the same family. The mean age of onset was 10 years and of diagnosis 26 years. Fifty (98%) patients were symptomatic, and 28 (55%) had experienced laryngeal edema. Deaths by laryngeal edema had occurred in 6 families. Crisis triggering factors were identified in 44 (86%) patients. Forty-five (88%) patients presented HAE due to quantitative deficit of C1-INH. Maintenance treatment was required for 32 (63%) patients, of whom 26 (81%) used Danazol, 5 (16%) Tranexamic acid, and 1 (3%) both. Thirteen (28%) patients needed icatibant to treat 23 crises.

## Conclusions

The diagnosis of HAE is still late and deaths due to severe attacks continue to occur. Therefore, it is important that health professionals are able to recognise and diagnose the disease and treat patients appropriately, as well as providing pharmacological services to control the disease.

